# The significance of heterophasic ion exchange in active biomonitoring of heavy metal pollution of surface waters

**DOI:** 10.1038/s41598-023-43454-7

**Published:** 2023-10-01

**Authors:** Andrzej Kłos, Sławomir Wierzba, Paweł Świsłowski, Agnieszka Cygan, Łukasz Gruss, Mirosław Wiatkowski, Krzysztof Pulikowski, Zbigniew Ziembik, Agnieszka Dołhańczuk-Śródka, Małgorzata Rajfur, Dominik Jerz, Magdalena Piechaczek-Wereszczyńska, Czesława Rosik-Dulewska, Piotr Wieczorek

**Affiliations:** 1 Institute of Environmental Engineering and Biotechnology, University of Opole, Kard. B. Kominka 6a, 45-032 Opole, Poland; 2 Institute of Biology, University of Opole, Oleska 22, 45-052 Opole, Poland; 3 Lukasiewicz - Institute of Ceramics and Building Materials, Environmental Engineering Division in Opole, Oświęcimska 21, 45-651 Opole, Poland; 4 Faculty of Chemistry, Department of Analytical Chemistry, Opole University, Oleska 48, 45-052 Opole, Poland; 5 Institute of Environmental Engineering, Wrocław University of Environmental and Life Sciences, Grunwaldzki Square 24, 50-363 Wrocław, Poland; 6 Institute of Environmental Engineering of the Polish Academy of Sciences, Skłodowskiej-Curie St. 34, 41-819 Zabrze, Poland

**Keywords:** Environmental biotechnology, Environmental monitoring, Pollution remediation

## Abstract

We have carried out studies to examine the possibility of using biosorbents: the epigeic mosses *Pleurozium schreberi* (Willd. ex Brid.) Mitt., and the epiphytic lichens *Hypogymnia physodes* (L.) Nyl. in active biomonitoring of heavy metal pollution of surface waters. The dried sea algae *Palmaria palmata* (L.) Weber & Mohr were used as the third biosorbent. The studies were conducted in the waters of the Turawa Reservoir, a dam reservoir with a significant level of eutrophication in south-western Poland. Incremental concentrations of Mn, Ni, Zn, Cu, Cd, and Pb were determined in the exposed samples. It was shown that a 2-h exposure period increases the concentration of some metals in the exposed samples, even by as much as several hundred percent. High increments of nickel concentrations in the algae *Palmaria palmata* (mean: 0.0040 mg/g, with the initial concentration of *c*_0_ < 0.0016 in the algae) were noted, with negligible increments in concentrations of this metal in mosses and lichens. In contrast, mosses and lichens accumulated relatively high amounts of Cd (mean: 0.0033 mg/g, *c*_0_ = 0.00043 mg/g) and Pb (mean: 0.0243 mg/g, *c*_0_ = 0.0103 mg/g), respectively.

## Introduction

The use of living organisms to assess environmental pollution became popular in the second half of the twentieth century. One of the first studies on biomonitoring of the aquatic environment was carried out in the early 1950s^1^. The following years saw a rapid growth of studies focusing on methods of air, water and soil biomonitoring. As a result, these methods have now supplemented programmes that protect, improve and control the quality of the environment.

In European Union countries, in accordance with the Directive of the European Parliament and of the Council No. 2000/60/EC of 23 October 2000, known as the Water Framework Directive, the principles of activities in the field of water management were established, including activities related to the monitoring of surface waters, where the basic criterion is the assessment of the status and ecological potential of waters. These studies are increasingly supplemented with biomonitoring studies related to the assessment of the chemical composition, mainly the concentrations of heavy metals. For quantitative studies, hydrophytes, e.g.: algae^2^, aquatic mosses^3^ and aquatic vascular plants, e.g. sea-grass^4^ are most commonly used. In addition, elements and chemical substances are determined in animal organisms, inter alia: in mussels^5^, aquatic snails^6^, crustaceans^7^, fish and their internal organs^8^. An overview of techniques and examples of the use of various organisms in biomonitoring of surface water pollution are presented in^9^ and in^10^. A detailed review of the applied biomonitoring methods and techniques that use aquatic mosses^11^ and algae has also been carried out^1^.

In passive biomonitoring techniques, which consist in the analysis of the chemical composition of organisms naturally present in a given water body and active biomonitoring techniques, which involve the exposure of organisms transferred from slightly polluted waters to the studied bodies of water, are used. In recent decades, plants (biosorbents) specially cultivated for this purpose have increasingly been used in active biomonitoring^12^. There are also ongoing studies focusing on the sorption properties of living and dead (dried) plants, e.g. aquatic mosses *Platyhypnidium riparioides*^13^, including using biosorbents that have been prepared chemically (e.g. extracted with EDTA to increase the sensitivity of the method)^14^ or thermally^15^. Numerous studies focus on optimizing the techniques for preparing samples intended for exposure^12^. After a period of exposure, increments in pollutants within the biosorbents are examined, and these can be considered proportional to their concentrations in water.

The authors agree that, during the first stage of biosorption of heavy metal cations, active centres of the extracellular structure are filled due to heterophasic ion exchange. The next stage is the transport of metal cations into the intracellular structure^16^.

In active biomonitoring of surface waters, the equilibrium between the heavy metal concentrations in the studied waters and their concentrations in the extracellular and intracellular structure of the biosorbent has been assumed as the state of equilibrium. The thus-interpreted equilibrium is reached after a few to several tens of days^12^, or even up to several months^10,17^, but there have been experiments that lasted shorter periods of time, ranging from 1 h to 7 days^18^.

A thesis was put forward (1) that the assessment of near-equilibrium of heterophasic ion exchange can constitute the basis for determining the distribution of heavy-metal cation concentrations in the studied waters, and for indicating their sources. The correctness of this thesis is demonstrated by the results of the authors’ own studies^19^ and those of other authors on the physicochemistry of such biosorption processes, indicating that the main sorption process (i.e. ion exchange between the extracellular structure and an aqueous solution) occurs with a 90% efficiency within approx. 60 min (e.g. in lichens: up to 60 min^20^; in mosses: up to 60 min^21^; and in sea algae: up to 35 min^22^). Furthermore, it was found that, after this time, not more than 10% of the total number of sorbed metals were accumulated in the intracellular structure^16^.

The thesis put forward suggests the possibility of using living or dead biosorbents that are not necessarily related to the environment of freshwater ecosystems and is extended by the further thesis (2) that: under the conditions of the experiment (pH, the ionic composition of the waters), the validity of the results can be improved by selecting biosorbents demonstrating sorption preferences towards the analysed heavy metal cations. For example, it was found^23^ that epigeic mosses accumulate greater amounts of Al, Ca, Cr and Ni, whereas epiphytic lichens favour Cu, Hg, Na, Ti and Zn. The authors’ own studies on biosorption of heavy metals from solutions demonstrate differences in the sorption preferences of various biosorbents^19^, including within a single division, e.g. mosses^24^. The preferential sorption of some metals to dead mosses biomass as compared to living biomass was also indicated^13^. It should be noted that the heavy metals biosorption efficiency is affected by the ionic composition of the studied waters^25^.

The aim of this study was to assess the possibility of using biosorbents, that are not naturally related to the environment of freshwater ecosystems, for the fast assessment of surface water pollution with heavy metals. The Big Turawa Reservoir (PL), a flow-through storage reservoir with a high level of eutrophication, was selected for the study.

The study of the distribution of heavy metal concentrations in the reservoir waters was preceded by studies of the kinetics of biosorption processes and by an assessment of the potential influence of pH, conductivity, and Na^+^, K^+^, Ca^2+^ and Mg^2+^ cation concentrations on the biosorption efficiency of the heavy metal cations. Changes in the biosorption efficiency of the heavy metal cations under the influence of changes in pH and salinity are demonstrated by other studies^25^.

The expediency of these studies results from the fact that the concentrations of heavy metals in surface waters are often lower than the quantification limits of other methods, e.g. atomic absorption spectrometry or mass spectrometry, which complicates how the experiment is conducted and significantly increases the cost of research. The proposed method of active biomonitoring, in comparison to other applied methods of active biomonitoring, significantly shortens the time of the experiment, from several/tens of days to two hours. The aspect of sorption preference of biosorbents with respect to specific metals is also important.

## Materials and methods

**Figure 1:**
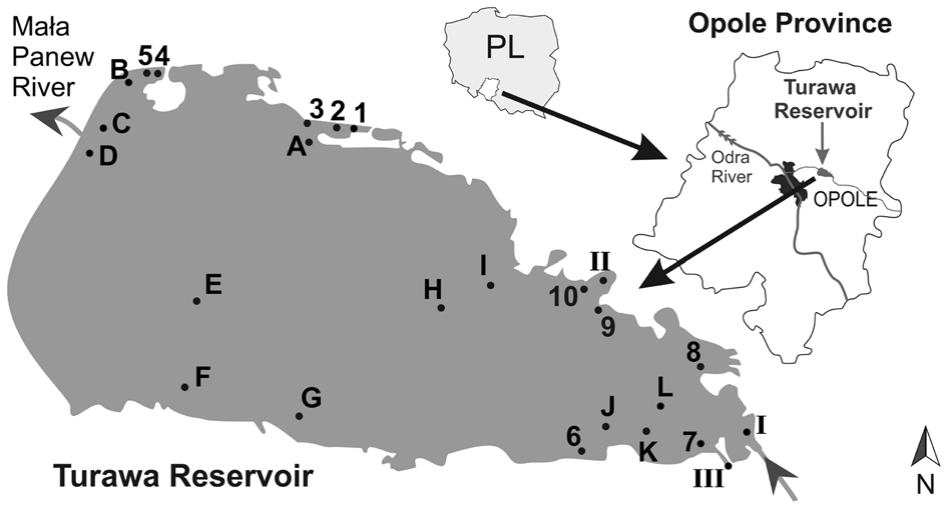
Location of the study area, detailing the sample exposure places.

The research was carried out in the waters of the Turawa Reservoir, located about 20 km north-east of the city of Opole (Fig. 1). This is a multi-purpose retention reservoir, built in 1933–39, with a maximum capacity of approximately 100 million cubic metres. The reservoir is fed mainly by the waters of the Mała Panew River which, apart from large quantities of biogenic substances causing eutrophication of the reservoir, also supplies into the reservoir heavy metals originating from catchment areas covering the industrialized areas of Upper Silesia. The significant heavy metal pollution of the Mała Panew River and its tributaries, including the Stoła river, is evidenced by the contamination of bottom sediments. In the years 2000–2002, special studies were carried out which showed that the bottom sediments (surface fraction < 1 mm) of the Stoła river and its Graniczna Woda tributary contained up to 3 mg g^−1^ Cd, 0.9 mg g^−1^ Cu, 20 mg g^−1^ Pb, and 30 mg g^−1^ Zn^26^. The reservoir also gathers water from the small Libawa and Rosa streams, and leachates pumped from fields in Jedlice and Szczedrzyk. For at least two decades, the Turawa Reservoir has been the subject of research into the causes and consequences of pollution, as well as on the possibilities of revitalizing it**.**

For the study, heavy metals were selected: Mn, Ni, Cu, Zn, Cd and Pb, whose main source of origin is the previously mentioned tributaries of the Mała Panew River from the industrial areas of Upper Silesia. Large amounts of these metals have accumulated in the bottom sediments over the years, constituting a secondary source of water pollution. Reports from the Voivodeship Inspector of Environmental Protection in Opole also indicate exceedances of Zn and Cd concentrations in the reservoir and in the waters of the catchment area.

### Determination of Na^+^, K^+^, Ca^2+^***and Mg***^2+^ concentrations, pH, and conductivity of the reservoir waters

The determination of Na^+^, K^+^, Ca^2+^ and Mg^2+^ cations was carried out in accordance with the standard for determination performed by ion chromatography: PN-EN ISO 10,304–1:2009/AC:2012 and PN-EN ISO 14,911:2002. Water samples for the determination of ionic composition were collected in accordance with the standard PN-EN ISO 5667–4:2003. Water pH and conductivity were measured in situ during the exposure of the biosorbent samples.

### Preparation, and manner and locations of exposure of the biosorbent samples

Verified biological materials characterized by good heavy metal sorption properties were selected for the studies^19^: mosses *Pleurozium schreberi* (Willd. ex Brid.) Mitt., lichens *Hypogymnia physodes* (L.) Nyl. and sea algae *Palmaria palmata* (L.) Weber & Mohr. Samples of the mosses and lichens were collected at one site, from ecologically clean areas in the Świętokrzyskie Province (PL). Dried sea algae were purchased from the company, Bogutyn Młyn, from Radzyń Podlaski (PL). The moss (green parts), lichens and algae samples were cleansed of mechanical impurities, rinsed with demineralized water (conductivity κ = 0.8 µS cm^−1^), and then conditioned in demineralized water for 5 h, each species separately. After conditioning, the samples were dried at a temperature of 303 K. About 300 g of dry mass of biosorbents intended for exposure were obtained. The initial concentrations *c*_0_ of the tested heavy metals Mn, Ni, Cu, Zn, Cd and Pb were determined in the prepared biosorbents over 5 repetitions. The biosorbents were stored in tightly closed polyethylene containers.

Samples for exposure, weighing about 2 g, were placed in nylon nets. These nets were attached to a nylon rope stretched between a weight falling to the bottom of the reservoir and a polystyrene block floating on the surface. The nets were placed 30 cm from the bottom of the reservoir and below the water surface. The exposure time was 2 h. After the exposure time, the samples were collected in polyethylene containers and dried at a temperature of 303 K. After drying the samples, to dry mass, the concentrations of the tested metals were determined. The samples were exposed in two periods: between August and November 2019 (where average values of the results from this period were analysed) and in May 2020. The exposure locations and the study area are shown in Fig. 1. At locations where the water depth was less than 0.5 m only one sample was exposed below the water surface. The maximum reservoir depth at the exposure locations was around 10 m for locations C–E, while the average depth did not exceed 4 m.

Figure 1 shows the selected exposure and sampling locations: in the littoral zone (locations 1–10), in the open water zone (locations A–L) and at the tributaries of the Mała Panew River (site I), the Libawa River (site II) and the Jedlice channel waters (site III).

In May 2020, at the sample exposure locations, the concentrations of cations: Na^+^, K^+^, Ca^2+^ and Mg^2+^ were also determined, as well as the pH value of the water samples collected below the reservoir surface.

### Apparatus and reagents

The presence of heavy metals Mn, Ni, Cu, Zn, Cd, and Pb were determined by flame atomic absorption spectrometry (FAAS) using iCE 3000 apparatus manufactured by Thermo Electron Corporation, USA. The samples were mineralized in a microwave mineralizer supplied by Berghof (D).

A Metrohm 850 Professional IC ion chromatograph was used to test the concentration of Na^+^, K^+^, Ca^2+^ and Mg^2+^ cations.

A pH-meter CP551, manufactured by Elmetron from Zabrze (PL), was used to determine the pH of the water samples. Its absolute error was: ∆pH = 0.02.

MERCK reagents were used for the tests.

### Quality assurance and control

Table 1 shows the limits of detection (IDL) and limits of quantification (IQL) of the iCE 3000 apparatus, as well as the limits of detection (MDL) and quantification (MQL) of the AAS analytical method determined on the basis of the results of metal determinations in homogenized and mineralized biosorbent samples. The values of MDL and MQL were determined assuming that biosorbent mass = 0.8 g d.m., solution volume after mineralization 25 mL.

**Table 1:** *IDL* and *IQL* values (mg L^−1^) and *MDL* and *MQL* values (mg g^−1^) converted to 1 g of dry mass of biosorbent.

Metal:	Mn	Ni	Cu	Zn	Cd	Pb
*IDL* (mg L^−1^)	0.0016	0.0043	0.0045	0.0033	0.0028	0.013
*IQL* (mg L^−1^)	0.010	0.024	0.015	0.010	0.010	0.044
*MDL* (mg g^−1^)	0.00101	0.00103	0.00088	0.00030	0.00026	0.00137
*MQL* (mg g^−1^)	0.0015	0.0016	0.0010	0.00050	0.00040	0.0022

Details of the determination of heavy metals in the certified reference material and the results of interlaboratory studies are shown in:^19^. In the data analysis, it was assumed that the uncertainty of the measurements of the tested metals in post-exposure samples was ± 20%. These are the highest uncertainty values of heavy metals measurements, determined with the AAS method in environmental samples, accepted in accredited research laboratories. For example, the uncertainties of the results of own research regarding the concentration of metals in homogenized moss samples and the uncertainties of the results of tests performed on the same material in an accredited laboratory (Zdravotní ústav se sídlem v Hradci Králové (CZ)) were Mn 8.8%, Ni 7.5%, Cu 3.9%, Zn 9.3%, Cd 10.3% and Pb 10.5%.

Table 2 presents data on the limits of detection and quantification of the chromatograph, and the uncertainty of the method’s measurement results, expressed by the standard deviation *s*_M_.

**Table 2:** Limits of detection (IDL) and quantification (IQL) and measurement uncertainty expressed by the standard deviation *s*_M_, characterizing the Metrohm 850 Professional IC ion chromatograph Metrohm 850 Professional IC.

Ion	*IDL* (mg L^−1^)	*IQL* (mg L^−1^)	*s*_M_ (%)
Na^+^	0.05	0.50	7
K^+^	0.05	0.50	19
Ca^2+^	0.1	1.0	9
Mg^2+^	0.1	1.0	11

### Method of interpretation and statistical assessment of results

The pseudo-second-order reaction model was used to assess the progress of heterophasic ion exchange after *t*^27^:

1 $$t/c_{{{\text{1}},{\text{t}}}} = {\text{ 1}}/k^{\hbox{''}} \cdot \left( {c_{{{\text{1}},{\text{e}}}} } \right)^{{\text{2}}} + {\text{ }}\left( {{\text{1}}/c_{{{\text{1}},{\text{e}}}} } \right) \cdot t$$

where: *k”*—pseudo-second-order reaction rate constant, *c*_1,e_—metal concentration in the biosorbent at equilibrium, *c*_1,*t*_—metal concentration in the biosorbent after *t*. This model allows differences between concentrations of metals in the biosorbent to be assessed after the period of exposure and the concentration of metals in the biosorbent at heterophasic equilibrium of ion exchange. On the basis of the model, it is possible to calculate the progress of the reaction after *t*: *c*_1,*t*_ and determine the percentage progress of the reaction:

2 $$P = { 1}00 \, \% \cdot \left( {c_{{{1},{\text{t}}}} /c_{{{1},{\text{e}}}} } \right)$$

The following relationship was used to analyse the influence of pH on the precipitation of hydroxides in the analysed metals:

3 $${\text{pH}}_{{{\text{prec}}.}} = z^{{ - {1}}} \cdot \, \left( {{14 } \cdot z{-}{\text{ log}}c_{M} {-}{\text{ p}}K_{{{\text{so}}}} } \right)$$

where: *z*—cation valency, *c*_*M*_—cation concentration in the solution (mol L^-1^), *K*_so_—solubility product; p*K*_so_ = −log(*K*_so_).

To evaluate changes in metal concentrations in the biosorbent immersed in water, a simple null hypothesis about the probability of success in a Bernoulli experiment was verified. The null hypothesis H_0_ stated that there was an average 0.5 probability of the metal concentration in the biosorbent increasing after it was immersed in water. The probability of the concentration decreasing (or remaining unchanged) is also 0.5. Confirming H_0_ would indicate that immersion does not affect the metal concentration in the biosorbent. The limits of 95% confidence intervals (CI) were also calculated. In the event that H_0_ was rejected, the position of CI with respect to *p* = 0.5 was analysed. An upper CI limit below 0.5 implies a decrease in the metal concentration. Conversely, a lower CI limit exceeding 0.5 confirms an increase in the metal concentration.

The impact effect of the tested factors on the metal concentration in the biosorbent was estimated. The impacts that biosorbent plant (*bs*:{*psch*, *hphy*, *ppal*}), season (*sz*:{*autumn*, *spring*}), water depth (*wd*:{*top*, *bottom*}) and water region (*wr*:{*nearshore*, *open-water*}) had on the metal concentration in the biosorbent were analysed. The plant material was immersed in water at the site identified according to the *id*. In order to estimate the statistical significance of the parameter affecting the metal concentration *c*_*met*_, a linear model was used, as described by the symbolic expression^28^:

4 $${\text{log}}\left( {c_{{{\text{met}}}} } \right) \, \sim id + bs + sz + wd + wr$$

where all the explanatory variables are factors.

The structural parameters β_*par*_ (*par*:{*id*, *bs*, *sz*, *wd*, *wr*}) in the model and the corresponding standard errors were calculated. The calculations were carried out in R language environment^29^. The actual parameters in the linear model were chosen using the selection procedure implemented in step() function, available in R. This function minimizes the AIC (Akaike Information Criterion) for the selection of the adequate subset of explanatory variables. For the selected variables, the corresponding *p*-values for the null hypothesis β_*par*_ = 0 were calculated. Additionally, for *p*-value > 0.05, the null hypothesis was not rejected, and the appropriate variable was removed from the actual model.

The results of the biomonitoring study were interpreted by comparing the increments ∆*c’* (mg g^˗1^) of metal concentrations in mosses, lichens and algae samples after the exposure period, minus the complex uncertainty of the expression: *c*_1_–*c*_0_. Only those results were considered which fulfilled Relationship:

5 $$\Delta {c}{\prime}={(c}_{1}-{c}_{0})-({c}_{1}\cdot 0.2+{s}_{{c}_{0}})>0$$

where: *c*_1_—concentration of metals after the exposure period, *c*_0_—concentration of metals before the exposure period, 0.2—coefficient resulting from the assumed measurement uncertainty *c*_1_ (± 20%), *s*_c0_—uncertainty of the initial concentration measurements in the exposed samples expressed by the standard deviation. The values of *c*_0_ and *s*_c0_ are presented in Table 3. For calculation of the concentration values *c* < *MQL, c* was assumed as *c* = 0.5 *MQL*.

**Table 3:** Initial metal concentrations *c*_0_ and the standard deviation values (mg g^−1^) in samples of mosses, lichens and algae intended for exposure in the waters of the Turawa Reservoir.

Biosorbent	Mn		Ni		Cu	
	*c*_0_	*s*_c0_	*c*_0_	*s*_c0_	*c*_0_	*s*_c0_
Mosses	0.155	0.013	< *MQL*	–	0.00652	0.00056
Lichens	0.135	0.011	< *MQL*	–	0.00504	0.00043
Algae	0.666	0.055	< *MQL*	–	0.00468	0.00046
Biosorbent	Zn		Cd		Pb	
	*c*_0_	*s*_c0_	*c*_0_	*s*_c0_	*c*_0_	*s*_c0_
Mosses	0.0388	0.0032	0.000428	0.000028	0.0069	0.0014
Lichens	0.0907	0.0064	0.000825	0.000032	0.0103	0.0011
Algae	0.0206	0.0014	< *MQL*	–	0.0108	0.0015

The results of the concentration measurements of cations Na^+^, K^+^, Ca^2+^ and Mg^2+^ as well as the pH of the water samples were interpreted by comparing their absolute values.

Box plots were used to visualize the increments of heavy metal concentrations in the exposed samples, indicating the median value, the minimum and maximum values, the upper and lower quartile, and outliers.

## Results and discussion

The interpretation of results of the biosorption efficiency studies and the biomonitoring studies was preceded by an analysis of data from reference sources regarding the kinetics of biosorption processes in extracellular and intracellular structures, and an analysis of the variability of cation concentrations, water pH and conductivity during the biosorbent exposure. These parameters may affect the biosorption of heavy metals from aqueous solutions^25^.

### Kinetics of biosorption processes in the extracellular and intracellular structure

In order to demonstrate the progress of the heterophasic ion exchange reaction during a 2-h exposure of biosorbents in heavy metal solutions, several examples of results of studies published by other authors regarding the biosorption of various metals from aqueous solutions were used. These studies apply the pseudo-second-order model (Relationship 1) to determine parameters of the kinetics of heterophasic ion exchange: *c*_1,e_ i *k*”. On their basis, using Relationship 1, it is possible to calculate the progress of the reaction after *t* (Relationship 2). The results calculated for 30, 60 and 120 min of exposure are presented in Table 4.

**Table 4:** Kinetic parameters of the heterophasic ion exchange reaction (Relationship 1) with the determined progress of the reaction (Relationship 2).

Metal/*c*_0,s_ (mg L^−1^)	*c*_1,e_ (mg g^−1^)		*k*" (g (mg min)^−1^)	*c*_1,*t*_ (mg g^−1^)		*P* (%)		
*t* (min) =			30	60	120	30	60	120
Biosorbent: moss *Sphagnum* sp.^27^								
Cu/25	5.75	0.0964	5.42	5.58	5.66	94	97	99
Ni/10	2.14	0.175	1.97	2.05	2.09	92	92	96
Pb/35	4.34	0.919	4.30	4.32	4.33	99	100	100
Biosorbent: alga *Fucus vesiculosus*^30^								
Cu/5	4.68	0.0158	3.23	3.82	4.21	69	82	90
Biosorbent: moss *Hylocomium splendens*^31^								
Cd/10	1.03	0.52	0.97	1.00	1.01	94	97	99
Biosorbent: moss *Drepanocladus revolvens*^32^								
Hg/10	1.99	0.086	1.67	1.81	1.90	84	91	95
Biosorbent: brewer’s spent grain^33^								
Cu/1,1	0.54	0.268	0.44	0.49	0.51	81	90	95

The presented results show that, after 120 min of exposure in solutions of the analysed metal salts, the process of heterophasic ion exchange occurs with an efficiency of at least 90%. In order to compare it with the live biomass, the table also presents results of copper sorption studies on brewer’s spent grain (barley meal), a waste product of the brewing industry^33^. Here, too, the progress of the heterophasic ion exchange process exceeds 90% after a 2-h exposure, indicating that this exchange occurs on the surface of both living and dead biomass.

As mentioned in the introduction, active biomonitoring of waters contaminated with heavy metals is usually carried out in a period of time that allows the extracellular and intracellular structure of living biosorbents to fill (i.e., from a few to several dozen of days). However, as indicated by the data in Table 4 and the data from reference sources^16^, the increments in metal concentrations in the biosorbents after long-term exposure do not exceed the accepted uncertainty of the measurement method: ± 20% for a 2-h exposure. The long-term exposure also has its drawbacks. It is difficult to determine the actual timing of maximum increments in concentrations, especially as regards river biomonitoring^34^. It is also worth noting that, with prolonged exposure times, the physiochemical and biotic factors can interfere with the biosorption process, causing the biological matter to b destroyed by, for example, decay, which in turn limits the use of dead biosorbents.

Therefore, the proposed 2-h exposure takes into consideration only the increments in metal concentrations in the biosorbents due to the ion exchange in the extracellular structure and assumes insignificant increments in metal concentrations in the intracellular structure. It does, however, allow for the appropriate selection of biosorbents (including dead ones) in terms of sorption preferences which, in turn, corresponds to the content of the proposed thesis (2) that the validity of the results can be improved by selecting biosorbents demonstrating sorption preferences towards the analysed heavy metal cations.

### ***Influence of Na***^+^***, K***^+^***, Ca***^***2***+^***and Mg***^***2***+^***cation concentrations, pH, and conductivity on biosorption efficiency***

One of the factors influencing the biosorption processes of heavy metal cations from aqueous solutions is the salinity of the waters, caused by the presence of cations Na^+^, K^+^, Ca^2+^ and Mg^2+^ and the pH of water. The results of studies on the sorption of heavy metals from aqueous solutions indicate that the biosorption of heavy metals is limited in particular by the Ca^2+^ cations present in waters and, in the case of acidic waters, hydrogen cations^35,36^.

In order to assess the influence of these factors, water samples were collected at the biosorbent exposure sites to determine Na^+^, K^+^, Ca^2+^ and Mg^2+^ cation concentrations and to measure water pH and conductivity in situ. Statistical parameters for the distribution of cation concentrations (mg L^−1^), and pH (-) and water conductivity κ (µS cm^−1^) are shown in Table 5.

**Table 5:** The statistical parameters of distribution of cation concentrations (mg L^−1^), pH values and conductivity of water at the exposure places of the biosorbent samples.

Parameter	Na^+^	K^+^	Ca^2+^	Mg^2+^	pH (-)	κ (μS cm^−1^)
Mean	23.6	9.5	51.1	8.5	7.5	433
± s	2.9	0.4	3.3	0.3	0.5	34
Min	19.8	8.7	40.9	8.0	6.6	398
Max	29.8	10.5	56.7	9.0	8.2	517

Laboratory studies conducted on the effect of Na^+^ (ca. 23 mg L^−1^) and Ca^2+^ (ca. 20 mg L^−1^) cations on the sorption of zinc cations from aqueous solutions in *Palmaria palmata* algae showed approx. 30% reduction in sorption efficiency, but with respect to the sorption of zinc cations from solutions not containing Na^+^ and Ca^2+^ admixtures. Furthermore, it was shown that, in the range of conductivity changes of 300–700 µS cm^−1^, the differences in sorption efficiency are within the limits of the measurement uncertainty^25^. The authors also indicate a reduction in the efficiency of heavy metals sorption from alkaline aqueous solutions^37^. It is indicated that this may be due to reactions leading to the formation of heavy metal hydroxocomplexes that, in extreme cases, cause precipitation of heavy metals in the form of poorly soluble hydroxides. Based on Relationship 3, it is possible to determine the concentration values above which, with the given pH, hydroxide precipitation occurs^19^. Taking into account the values of solubility equilibrium^38^ and data on the content of heavy metals in the waters of the Mała Panew River feeding the reservoir: Ni < 10 µg L^−1^, Cd ≤ 10.6 µg L^−1^, Pb ≤ 10.4 µg L^−1^^39^, it can be concluded that, at the maximum recorded pH = 8.2 (Table 5), the concentrations of Ni and Cd are at least 3 orders of magnitude lower than the concentrations initiating precipitation of hydroxides. Only the maximum Pb concentrations were of the same order.

Therefore, it should be assumed that, given the demonstrated variability in concentrations of the cations in question, the differences in pH and water conductivity values in the Turawa Reservoir do not significantly affect the differences in the biosorption conditions of the studied heavy metals at the designated exposure locations. The heterophase equilibria of biosorbent—solution and the kinetics of heavy metal sorption processes from aqueous solutions are also affected by temperature. It should be noted, however, that the studied water body is a shallow reservoir and temperature differences in the reservoir volume also do not significantly affect the sorption process kinetics and the significant differences in water density in hydrometric risers.

Therefore, the increments in the concentrations of the analysed metals in the biosorbents after the exposure period depend primarily on the concentration of the ionic forms of these metals in the water. Under the conditions of the experiment, possible sorption limitations caused by differences in ionic composition and pH of the water between the exposure sites of the biosorbent samples are lower than the assumed measurement uncertainty (± 20%).

### Biosorption of heavy metals in the Turawa Reservoir waters

The heavy metal biosorption results were examined in terms of differences due to exposure sites and biosorbent types, differences in the distribution of increments in metal concentrations in the biosorbents (also taking into consideration the initial concentrations) and the differences in increments in metal concentrations, taking into account the assumed measurement uncertainty.

#### Distribution of metal concentrations in biosorbents after the exposure period

The statistical data (median, standard deviation, minimum, maximum) concerning the measurements of metal concentrations in the samples of mosses, lichens and algae after a 2-h exposure in the waters of the reservoir were collected in Table 6. The mean values and statistical parameters were determined for samples exposed near the bottom and just below the water surface in two periods from August to November 2019 and in May 2020.

**Table 6:** Mean values of metal concentrations (mg g^−1^) in samples of mosses (M), lichens (L) and algae (A) exposed in the waters of the Turawa Reservoir; ± *s*—standard deviation.

Parameter	Mn	Ni	Cu	Zn	Cd	Pb
Moss *Pleurozium schreberi* (M)						
Mean	0.1260	0.00098	0.0067	0.0589	0.0033	0.0101
± s	0.0580	0.00074	0.0042	0.0723	0.0093	0.0068
Min	0.0011	0.00080	0.0008	0.0062	0.0002	0.0004
Max	0.2929	0.00560	0.0375	0.4313	0.0678	0.0332
Lichen *Hypogymnia physodes* (L)						
Mean	0.0639	0.00090	0.0051	0.0761	0.0010	0.0243
± s	0.0432	0.00054	0.0019	0.0246	0.0006	0.0128
Min	0.0230	0.00080	0.0018	0.0244	0.0002	0.0089
Max	0.3020	0.00500	0.0188	0.1972	0.0043	0.0800
Algae *Palmaria palmata* (A)						
Mean	0.2943	0.0040	0.0076	0.0432	0.0008	0.0094
± s	0.5136	0.0018	0.0088	0.0301	0.0009	0.0089
Min	0.0100	0.0008	0.0028	0.0197	0.0002	0.0011
Max	2.9190	0.0139	0.0585	0.2163	0.0053	0.0683

Detailed measurement results are presented in the supplementary materials.

#### Analysis of selected factors influencing biosorption processes

The impact effect of the factors on the metal concentration in the biosorbent was estimated with respect to Eq. (4). The calculation results indicated that the impact effects on metal concentration differed between parameters. In the case of the Mn concentration, significant differences in relation to the sampling site, the biosorbent type and the season were noted. At most sites, the Mn concentrations were different. Higher Mn concentrations were determined in algae and mosses. Biosorbents immersed in water absorbed more Mn in the spring than in the autumn. The Ni concentration only depended on the biosorbent type. The metal concentrations in lichens and mosses were similar, whereas significantly higher Ni concentrations were observed in algae. The average Cu concentrations depend on the biosorbent type and the season. The Cu concentrations were higher in mosses and algae than in lichens. In the spring, the Cu concentration in biosorbents decreases. The Zn concentrations differed between sampling sites and were related to the biosorbent type. Apart from two exceptional sites, the distribution of concentrations in the reservoir area was fairly homogeneous. The Zn content was higher in lichens than in mosses and algae. The Cd distribution over the sampling sites is generally uniform, though three exceptions were noted. Differences in Cd concentration were also related to the biosorbent type. The main difference in comparison to other metals was that the Cd concentration was significantly higher in the bottom zone than in the surface zone. The Pb concentration was related only to the biosorbent type. In lichens, the Pb concentration was higher than in the other materials. Table 7 presents the summarized results in a concise form.

**Table 7:** Parameters influencing the metal concentration in the biosorbents. The “x” mark indicates statistically significant effect of the variable on the metal concentration.

Metal concentration/parameter	Mn	Ni	Cu	Zn	Cd	Pb
Site (*id*)	x			x	x	
Biosorbent (*bs*)	x	x	x	x	x	x
Season (*sz*)	x		x			
Water depth (*wd*)					x	
Water region (*wr*)						

The Mn, Zn and Cd concentrations were related to the site. For each metal, its accumulation depends on the biosorbent type. The season affects the Mn and Cu concentrations. Apart from Cd, no impact of water depth on metal concentration was noted. No impact of water region on metal accumulation was found.

#### Distribution of increments in metal concentrations in biosorbent samples after the exposure period

**Figure 2:**
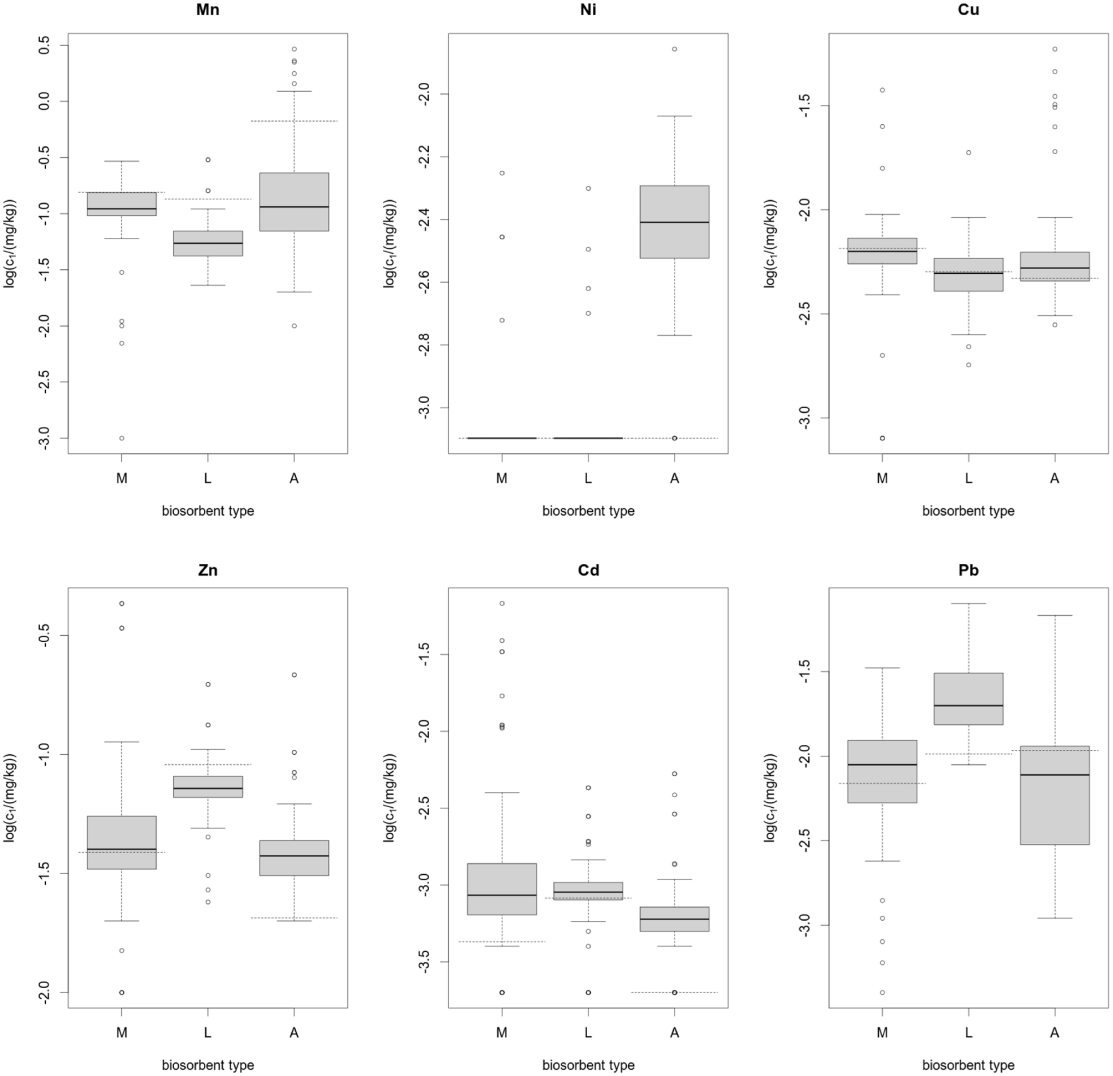
Visualization of the distribution of metal concentrations in samples of mosses (M), lichens (L) and algae (A) exposed in the waters of the Turawa Reservoir.

The distribution of metal concentrations in biosorbents after the period of exposure is visualized in Fig. 2. The distribution of variables in the logarithmic form (log*c*_1_) is demonstrated using box plots, showing the median value, the minimum and maximum values, the upper and lower quartiles, and outliers. The values log*c*_0_ are marked with a horizontal dashed line on the graphs.

Table 8 presents changes in metal concentration in the biosorbent with respect to initial c_0_. In the table, *n* refers to the number of trials (number of biosorbent samples immersed in water), *k* to the number of successes (number of samples with metal concentrations higher than c_0_), *p*-value to the test probability for H_0_: p_success_ = 0.5; CI_l_ and CI_r_—to the left and right limits of the p_success_ 95% confidence interval, respectively; whereas the impact effect describes the total change in the metal concentration in the biosorbent.

**Table 8:** Changes in the metal concentration in biosorbent in respect to the initial c_0_.

Metal	Biosorbent	*n*	*k*	*p*-value	CI_l_	CI_r_	Effect
Mn	Moss	94	21	0.000	0.144	0.321	Loss
Mn	Lichen	92	4	0.000	0.012	0.108	Loss
Mn	Alga	92	10	0.000	0.053	0.191	Loss
Ni	Moss	94	4	0.000	0.012	0.105	Loss
Ni	Lichen	92	4	0.000	0.012	0.108	Loss
Ni	Alga	92	87	0.000	0.878	0.982	Accumul
Cu	Moss	94	38	0.079	0.304	0.510	No change
Cu	Lichen	92	43	0.602	0.363	0.574	No change
Cu	Alga	92	67	0.000	0.626	0.816	Accumul
Zn	Moss	94	55	0.121	0.479	0.686	No change
Zn	Lichen	92	9	0.000	0.046	0.178	Loss
Zn	Alga	92	90	0.000	0.924	0.997	Accumul
Cd	Moss	94	78	0.000	0.738	0.899	Accumul
Cd	Lichen	92	57	0.028	0.512	0.719	Accumul
Cd	Alga	92	76	0.000	0.733	0.897	Accumul
Pb	Moss	94	62	0.003	0.555	0.754	Accumul
Pb	Lichen	92	89	0.000	0.908	0.993	Accumul
Pb	Alga	92	29	0.001	0.222	0.420	Loss

The results presented in Table 8 demonstrate that the changes in metal concentration in the material depend both on the metal and on the type of biosorbent, leading to the following conclusions: (1) regardless of the type of biosorbent, Mn concentration decreases after immersion of the material in water; (2) Ni concentration decreases in mosses and lichens, but increases in algae; (3) Cu concentration does not change in mosses and lichens, but increases in algae; (4) Zn concentration does not change in mosses, decreases in lichens, and increases in algae; (5) Cd concentration increases in mosses, lichens and algae, with the most increments (Table 8) and highest increments (Table 6) being noted in mosses; (6) Pb concentration increases in mosses and lichens, and decreases in algae.

#### Influence of the initial concentration of metals in biosorbents on their increments during exposure

A comparison of the data in Tables 3 and 6 shows that one of the factors resulting in high increments in metal concentrations in the exposed samples may be the low initial concentration *c*_0_ of metals in the biosorbents. This relationship is fulfilled for Cu and Zn. For Ni, Cd and Pb, the initial concentration does not clearly influence the sorption preferences. For Ni, the initial concentrations for all the biosorbents are below *MQL*. Mosses are characterized by the highest number of increments in Cd concentration. The initial Cd concentration is significantly higher in mosses than in algae. Also, in the case of Pb, the highest number of concentration increments was noted for lichens, whose *c*_0_ value is higher than that of mosses. As previously mentioned, there were no Mn increments in most of the exposed samples (Table 8).

#### Assessment of increments in metal concentrations after the exposure period, taking into consideration the measurement uncertainty

The next stage of the data analysis involved assessing the number of results that fulfil Relationship 5, taking into account the assumed uncertainty in the determination of the increments of the examined metals: Δ*c*’ > 0. Table 9 shows the percentage of samples for which relationship 5 was fulfilled with respect to the tested metals.

**Table 9:** Percentage (%) of samples for which relationship 3 was fulfilled.

Biosorbent	Mn	Ni	Cu	Zn	Cd	Pb
Moss	8.7	4.4	5.3	24.5	76.6	33.0
Lichen	2.2	4.2	7.6	4.3	20.6	77.2
Algae	6.5	92.4	23.9	80.4	64.1	12.0

For Ni and Zn, algae accounted for the highest number of samples with significant increments in concentrations. Lichen samples demonstrated the highest number of increments in Pb concentrations, whereas moss samples showed the highest number of increases in Cd concentrations. The number of samples with increments in Mn and Cu concentrations in all the biosorbents was insignificant, which was also initially demonstrated by the data in Table 6 and shown graphically in Fig. 2.

### Application of the method for assessing the distribution of heavy metal concentrations in the Turawa Reservoir waters

**Figure 3:**
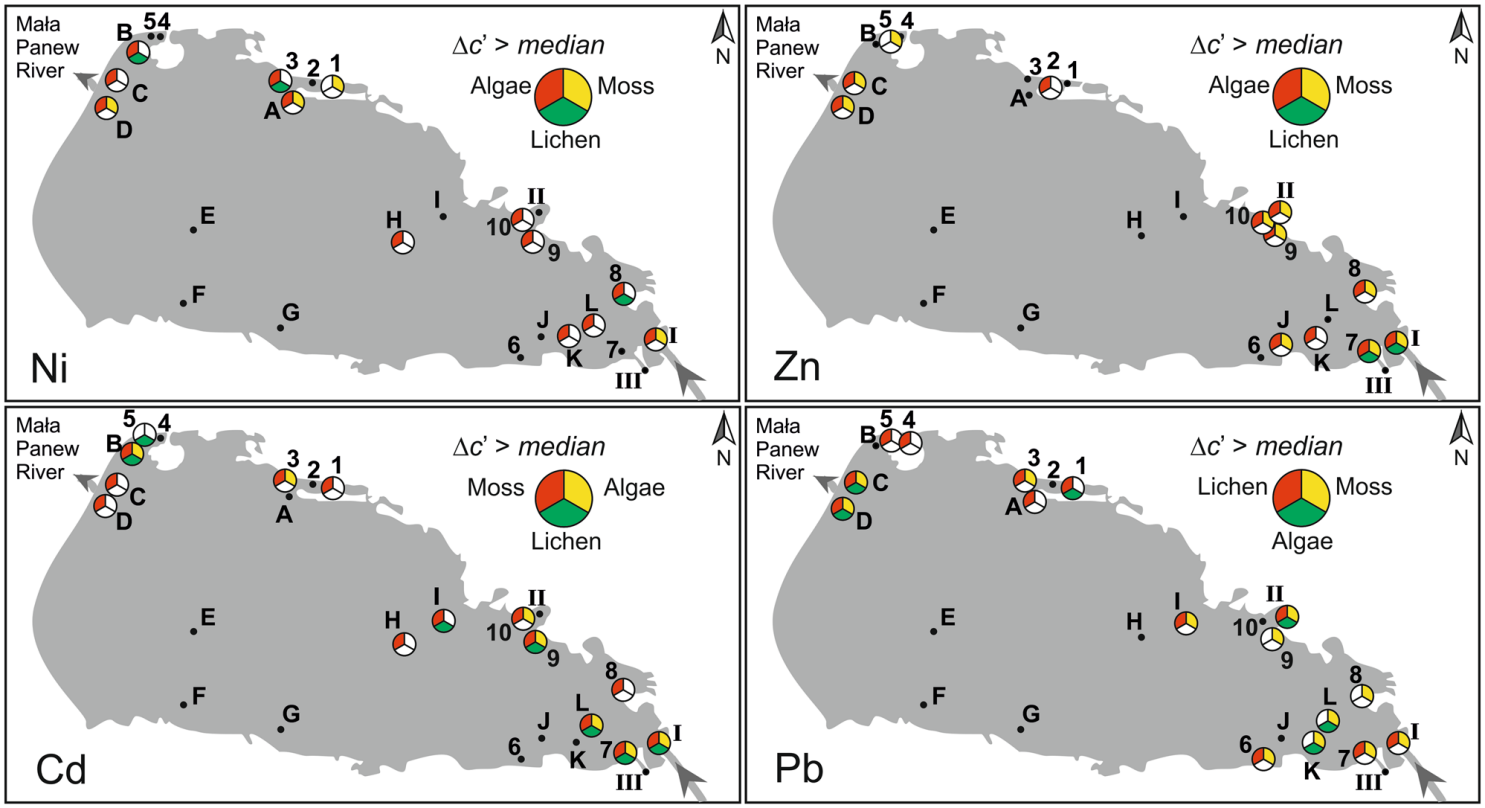
Distribution of increases in metal concentrations, defined by relationship 5, in mosses, lichens and algae exposed in waters of the Turawa Reservoir.

The described method and the collected data were used to evaluate the distribution of Ni, Zn, Cd and Pb concentrations in the waters of the Turawa Reservoir. Figure 3 lists the locations of exposure where the increments of concentrations of the tested metals ∆*c’* (Relationship 5) were greater than the median value determined for a given metal from all exposure locations. The Figure lists the biosorbents with the best sorption properties for a given metal in red and then in yellow and green, in descending order. White or no markings indicate values lower than, or equal to the median value.

The distribution of increases in metal concentrations in the exposed samples points to the primary source of pollution being the waters of the Mała Panew River, which is also confirmed by other studies^26,40^. On the other hand, the observed increases in metal concentrations in locations 1–5 and A-D are the result of secondary contamination of waters with heavy metals from bottom sediments. The accumulation of large amounts of sapropelic sediments in the northern part of the reservoir, especially at the outflow of the Mała Panew River is indicated by studies conducted in 2004^40^. The concentrations of Zn, Cd and Pb in the sediment samples were estimated to be respectively: 7000–8000 mg kg^−1^, 350–400 mg kg^−1^ and 600–700 mg kg^−1^. The results of the study published in 2011 also confirm high concentrations of heavy metals in sediments accumulated in the northern part of the reservoir: Ni (mean: 36.6 mg kg^−1^), Zn (mean: 4770 mg kg^−1^), Cd (mean: 280 mg kg^−1^), Pb (mean: 312 mg kg^−1^)^41^. Another secondary source of heavy metal contamination from sapropelic sediments was located near sites H and I^40^.

In addition to substantiating the distribution of heavy metals in the reservoir waters, the efficiency of the selected biosorbents was also compared in order to assess the validity of the proposed method. In the case of determination of Ni increments, only at site 1 were no increments in the preferred biosorbent (algae) noted, but the increments were recorded in mosses. For determination of Zn (algae) and Cd (moss), this discrepancy was also recorded only at one site, namely site 5. On the other hand, for lead (lichens), four such sites were found, namely sites 8, 9, K and L. However, these irregularities represent only 2% in relation to the product of the number of sites, the number of biosorbents used, and the number of metals determined.

A drawback of the proposed biomonitoring method, compared to classical methods of measuring concentrations of heavy metals in surface waters, is the lack of the possibility to convert the obtained results into absolute metal concentration values in the tested waters. However, based on the obtained results, it is possible to determine the sources and directions of the spreading of heavy metals in the tested waters.

## Conclusions

The presented study results demonstrate the possibility of simplifying the previously used examination procedures in the active biomonitoring of surface waters. This is associated with a significant reduction in the exposure time of the samples while maintaining comparable test validity. Regardless of the selected active biomonitoring technique, the assessment concerns the distribution of concentrations of the analysed metals, with no universal translation into the actual concentrations of these metals in the waters examined. Additionally, it has been shown that the validity of the results can be improved by the appropriate selection of biosorbents. Under the conditions of the experiment, the sea algae *Palmaria palmata* (L.) Weber & Mohr had the best sorption properties for nickel and zinc, with negligible increments in concentrations of the metals in mosses and lichens. Cadmium accumulated best in the epigeic mosses *Pleurozium schreberi* (Willd. ex Brid.) Mitt., and lead in the epiphytic lichens *Hypogymnia physodes* (L) Nyl.

Detailed analyses showed that: (1) after a 2-h exposure period, the progress of the ion exchange reaction exceeds 90%; (2) under the conditions of the experiment, the influence of the basic chemical parameters of the water (macrocation concentrations, water pH and conductivity) do not significantly influence the distribution of the concentrations of the examined heavy metals in the reservoir waters; (3) the distribution of the increments in the concentrations of the analysed metals after the exposure period depends mainly on the exposure site and the type of biosorbent; and (4) the increments in concentrations of the examined metals in the selected biosorbents were significantly higher than the assumed uncertainty (± 20%).

### Supplementary Information

Supplementary Information.

## Data Availability

The datasets generated during and/or analysed during the current study are available in the Mendeley Data repository, Mendeley Data, V1, https://doi.org/10.17632/3ksnrt75y5.1.
