# Author Correction: The significance of heterophasic ion exchange in active biomonitoring of heavy metal pollution of surface waters

**DOI:** 10.1038/s41598-023-47252-z

**Published:** 2023-11-16

**Authors:** Andrzej Kłos, Sławomir Wierzba, Paweł Świsłowski, Agnieszka Cygan, Łukasz Gruss, Mirosław Wiatkowski, Krzysztof Pulikowski, Zbigniew Ziembik, Agnieszka Dołhańczuk-Śródka, Małgorzata Rajfur, Dominik Jerz, Magdalena Piechaczek-Wereszczyńska, Czesława Rosik-Dulewska, Piotr Wieczorek

**Affiliations:** 1 Institute of Environmental Engineering and Biotechnology, University of Opole, Kard. B. Kominka 6a, 45-032 Opole, Poland; 2 Institute of Biology, University of Opole, Oleska 22, 45-052 Opole, Poland; 3 Lukasiewicz - Institute of Ceramics and Building Materials, Environmental Engineering Division in Opole, Oświęcimska 21, 45-651 Opole, Poland; 4 Faculty of Chemistry, Department of Analytical Chemistry, Opole University, Oleska 48, 45-052 Opole, Poland; 5 Institute of Environmental Engineering, Wrocław University of Environmental and Life Sciences, Grunwaldzki Square 24, 50-363 Wrocław, Poland; 6 Institute of Environmental Engineering of the Polish Academy of Sciences, Skłodowskiej-Curie St. 34, 41-819 Zabrze, Poland

Correction to: *Scientific Reports* https://doi.org/10.1038/s41598-023-43454-7, published online 01 October 2023

The Funding section in the original version of this Article was incomplete.

“Financial support for this work was provided by the National Centre for Research and Development, programme BIOSTRATEG (ZBIORTUR, project 3/343733/15/NCBR/2018).”

now reads:

“Financial support for this work was provided by the National Centre for Research and Development, programme BIOSTRATEG (ZBIORTUR, project 3/343733/15/NCBR/2018). The APC is co-financed by Wrocław University of Environmental and Life Sciences.”

The original Article has been corrected.

